# Novel ABCC2 mutations in a neonate with Dubin-Johnson syndrome: a case report

**DOI:** 10.3389/fendo.2026.1913891

**Published:** 2026-10-15

**Authors:** Lijun An, Yuanda Zhang, Sisi Zhang

**Affiliations:** 1 Department of Nutrition, Baoding Hospital, Beijing Children’s Hospital Affiliated to Capital Medical University, Baoding, Hebei, China; 2 Department of Gastroenterology, Baoding Hospital, Beijing Children’s Hospital Affiliated to Capital Medical University, Baoding, Hebei, China

**Keywords:** ABCC2 gene, Dubin-Johnson syndrome, neonatal-onset cholestasis, next-generation sequencing, novel mutation

## Abstract

**Background:**

Dubin-Johnson syndrome (DJS) is a rare autosomal recessive disorder caused by mutations in the *ABCC2* gene, leading to impaired excretion of conjugated bilirubin. Neonatal or infantile onset is uncommon and easily misdiagnosed as biliary atresia or progressive familial intrahepatic cholestasis.

**Case presentation:**

We report a female infant who presented with persistent jaundice since birth (onset in the neonatal period, ≤28 days). A definitive genetic diagnosis of Dubin-Johnson syndrome (DJS) was established at 11 months of age through next-generation sequencing. Her subsequent 5-year follow-up indicates a favorable clinical course. Laboratory tests revealed consistently normal transaminase levels with elevated total bilirubin (peak 120.3 μmol/L at 52 days), direct bilirubin (peak 61.3 μmol/L), and gamma-glutamyl transferase (88 U/L). Next-generation sequencing identified two novel compound heterozygous mutations in the *ABCC2* gene: c.2443C>T (p.R815X, nonsense) inherited from the mother and c.4465_4473delinsGGCCCACAG (p.1489_1491delinsGPQ, in-frame) inherited from the father. Both mutations were classified as pathogenic/likely pathogenic according to ACMG guidelines (c.2443C>T: PVS1, PM2, PM3; c.4465_4473delinsGGCCCACAG: PM2, PM3, PM4) and have not been previously reported. Ursodeoxycholic acid therapy was administered, and bilirubin levels showed progressive decline over time; however, this improvement may also reflect the spontaneous trend toward resolution that is characteristic of the natural history of DJS.

**Conclusion:**

This case expands the mutational spectrum of *ABCC2* gene and highlights the critical role of genetic testing in early diagnosis of infantile DJS, thereby avoiding unnecessary invasive procedures. The favorable prognosis with conservative management further supports the benign nature of this condition.

## Introduction

Dubin-Johnson syndrome (DJS) is a rare autosomal recessive disorder caused by mutations in the *ABCC2* gene. Dysfunction of the encoded multidrug resistance-associated protein 2 (MRP2) impairs the excretion of conjugated bilirubin from hepatocytes into the biliary system, resulting in recurrent episodes of predominantly conjugated hyperbilirubinemia as the major clinical manifestation (1). DJS most commonly presents during adolescence, whereas neonatal or infantile onset is uncommon and may be misdiagnosed as more severe cholestatic disorders, including biliary atresia and progressive familial intrahepatic cholestasis (PFIC) (2, 3).

With the widespread use of next-generation sequencing (NGS), a growing number of neonatal DJS cases have been accurately identified through genetic analysis, thereby reducing the need for unnecessary invasive diagnostic procedures and interventions (4). Nevertheless, establishing a diagnosis during early infancy remains challenging because the clinical presentation is often nonspecific. Moreover, the characteristic melanin-like pigment deposition in liver tissue is detected in only approximately 38% of affected neonates (2). Given the limited diagnostic utility of its clinical manifestations and histopathology, genetic testing has emerged as an essential tool for confirming the diagnosis in this patient population. To date, more than 100 *ABCC2* variants have been reported worldwide, with the mutational spectrum demonstrating considerable ethnic and geographic heterogeneity (5).

In this report, we describe a female infant with persistent conjugated hyperbilirubinemia since birth. NGS identified compound heterozygous ABCC2 variants, c.2443C>T (p.R815X) and c.4465_4473delinsGGCCCACAG (p.1489_1491delinsGPQ), inherited from her mother and father, respectively. While the c.4465_4473delinsGGCCCACAG variant has been recently reported by Zhao C et al., the c.2443C>T (p.R815X) variant has not been previously described (5). By correlating the patient’s clinical characteristics, longitudinal biochemical findings, and genetic test results, this report aims to enhance recognition of DJS with neonatal onset and highlight the indispensable role of genetic testing in early diagnosis.

## Case presentation

A female infant, now 5 years of age, was referred to our hospital for evaluation of persistent jaundice that had been noted since birth. The diagnosis of DJS was ultimately confirmed at 11 months of age via genetic testing. This report details her clinical course from the neonatal period through to her current 5-year follow-up. Hyperbilirubinemia was noted shortly after birth, characterized predominantly by elevated direct bilirubin levels and accompanied by yellow discoloration of the skin and sclerae. Phototherapy was administered at a local hospital, resulting in temporary improvement in jaundice and serum bilirubin concentrations. However, hyperbilirubinemia recurred soon thereafter. At 52 days of age, the patient was admitted to the neonatal department for further evaluation. Liver biochemistry revealed an alanine aminotransferase (ALT) level of 17.4 U/L (reference: 5–40 U/L), aspartate aminotransferase (AST) of 36 U/L (5–40 U/L), total bilirubin (TBil) of 120.3 μmol/L (2–19 μmol/L), direct bilirubin (DBil) of 61.3 μmol/L (0–6.8 μmol/L), indirect bilirubin (IBil) of 59.0 μmol/L (1.7–13 μmol/L), gamma-glutamyl transpeptidase (GGT) of 88 U/L (5–50 U/L), and total bile acid (TBA) of 12.7 μmol/L (0–10 μmol/L). Notably, the indirect bilirubin fraction was also elevated (59.0 μmol/L), contributing to a mixed hyperbilirubinemia at initial presentation. A hemolysis screen, including reticulocyte count and peripheral blood film, was performed and showed no evidence of significant hemolysis. Direct antiglobulin (Coombs) testing was negative, excluding immune-mediated hemolysis. This indirect hyperbilirubinemia was therefore attributed to physiological neonatal jaundice combined with an element of bilirubin overproduction in the setting of cholestasis, rather than an independent hemolytic process. Following the initial presentation at 52 days of age, the patient was started on UDCA and closely monitored. Given the persistent conjugated hyperbilirubinemia with normal transaminases and the absence of biliary obstruction on imaging, a genetic etiology was suspected. At 11 months of age, peripheral blood samples from the proband and her parents were collected for next-generation sequencing (NGS) and subsequent Sanger validation, which led to the confirmatory genetic diagnosis. The timeline from initial presentation to final diagnosis spanned from day 52 to 11 months of life. Serial follow-up assessments demonstrated persistently normal ALT and AST levels. GGT gradually normalized, whereas TBil, DBil, IBil, and TBA remained above the reference ranges but showed a progressive downward trend over time. The patient was the first child born to healthy non-consanguineous parents following an uncomplicated full-term vaginal delivery. Growth and developmental milestones were appropriate for age. There was no known family history of hereditary disease. On physical examination, body temperature was 36.7 °C, pulse rate 100 beats/min, respiratory rate 24 breaths/min, blood pressure 102/64 mmHg, and body weight 18 kg. Mild scleral icterus and slight cutaneous yellowing were present. No palmar erythema or spider angiomas were observed. Kayser-Fleischer rings were absent. The abdomen was soft and non-tender, with no palpable hepatomegaly or splenomegaly. Cardiopulmonary and neurological examinations were unremarkable. Laboratory findings, including liver function parameters, are presented in **Figure 1** and **Table 1**. Complete blood count showed a white blood cell count of 8.43 × 10^9^/L, red blood cell count of 4.28 × 10¹²/L, platelet count of 310 × 10^9^/L, and hemoglobin concentration of 115 g/L. Stool color was monitored throughout the follow-up period and remained consistently pigmented (normal yellow-green color), with no acholic or pale stools observed. Routine urinalysis and stool examinations showed no abnormalities. Screening for hepatotropic viruses and viral hepatitis was negative. Both the direct antiglobulin test and Coombs test yielded negative results. Serum copper and ceruloplasmin concentrations were within normal limits. Thyroid function tests were also normal. Urinary coproporphyrin analysis was not performed in this patient. Abdominal color Doppler ultrasound revealed no structural abnormalities. Transient elastography showed a liver stiffness measurement of 4.4 kPa, which is within the normal range for healthy children (generally <5.0–5.5 kPa) and argues against significant hepatic fibrosis. Nuclear magnetic resonance imaging of the upper abdomen and magnetic resonance cholangiopancreatography were unremarkable. High-throughput sequencing was subsequently performed using NGS, followed by Sanger sequencing for parental validation. All variant descriptions followed the Human Genome Variation Society (HGVS) nomenclature guidelines, using the ABCC2 reference transcript NM_000392.5 as the reference sequence. Two heterozygous variants in the *ABCC2* gene were identified in the proband: c.2443C>T (a nonsense mutation resulting in p.R815X), inherited from the mother, and c.4465_4473delinsGGCCCACAG (an in-frame mutation resulting in p.1489_1491delinsGPQ), inherited from the father (**Figures 2**, **3**). Variant nomenclature follows HGVS recommendations based on the ABCC2 reference transcript NM_000392.5.According to the guidelines of the American College of Medical Genetics and Genomics (ACMG), c.2443C>T was classified as pathogenic, and c.4465_4473delinsGGCCCACAG was classified as likely pathogenic. The specific ACMG/AMP evidence codes applied were as follows: for the nonsense variant c.2443C>T (p.R815X), PVS1 (null variant in a gene where loss-of-function is a known mechanism of disease), PM2 (absent from controls in population databases), and PM3 (detected in trans with a pathogenic variant). For the in-frame indel variant c.4465_4473delinsGGCCCACAG (p.1489_1491delinsGPQ), PM2 (absent from controls), PM3 (detected in trans with a pathogenic variant), and PM4 (protein length change due to in-frame deletion/insertion in a non-repeat region) were applied. Both variants were found to be rare in healthy population databases. Both variants were found to be rare in healthy population databases. No functional mutations were detected in other genes associated with bilirubin transport and metabolism, including SLCO1B1, SLCO1B3, and UGT1A1. Considering the patient’s history of jaundice, repeated episodes of conjugated hyperbilirubinemia with a cholestatic pattern, persistently normal aminotransferase levels, mild jaundice without hepatosplenomegaly on physical examination, and the presence of compound heterozygous *ABCC2* variants, a diagnosis of DJS caused by *ABCC2* mutations was established. Following confirmation of the diagnosis, oral UDCA therapy was continued at a dose of 10–15 mg/kg/day without interruption. Treatment response was primarily gauged by serial bilirubin monitoring and clinical assessment of jaundice. Given the established genetic diagnosis and the patient’s favorable clinical course with conservative management, no liver biopsy was performed. Follow-up abdominal ultrasound at 5 years of age showed no evidence of hepatomegaly, splenomegaly, or biliary abnormalities. At the 1-month follow-up visit, the degree of skin and scleral yellowing had diminished, and bilirubin levels showed further improvement. The patient’s growth and developmental milestones were appropriate for her age, with current weight (18 kg) and height at the 50th–75th percentile for a 5-year-old female. The patient remains under regular follow-up. The clinical timeline of the patient spanning birth to the 5-year follow-up is presented in **Figure 4**.

**Figure 1:**
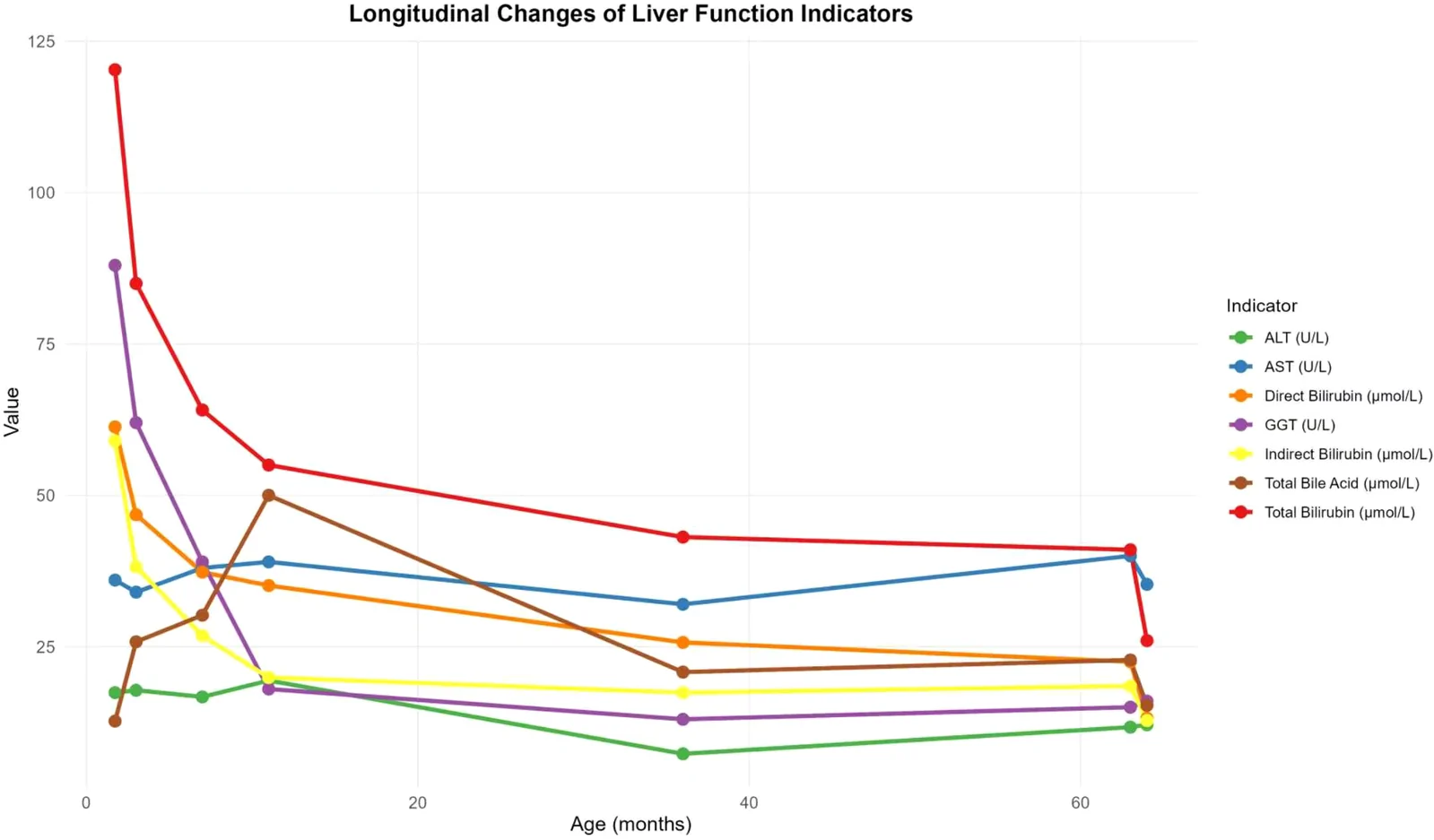
Longitudinal changes of bilirubin levels in the patient with Dubin-Johnson syndrome. The image illustrates the evolution of total bilirubin (Tbil), direct bilirubin (Dbil), and indirect bilirubin (Ibil) from 52 days to 5 years and 4 months of age. All three bilirubin fractions reached their maximum levels at 52 days (1.73 months) and exhibited a sustained downward trend thereafter, with the most rapid decline occurring within the first 11 months of life. Direct bilirubin accounted for more than 50% of total bilirubin at all time points, consistent with the conjugated hyperbilirubinemia characteristic of Dubin-Johnson syndrome.

**Table 1:** Serial liver function parameters from 52 days to 5 years and 4 months of age.

Liver function indicators	Age						
	52d	3m	7m	11m	3y	5y 3m	5y 4m
ALT(5~40 U/L)	17.4	17.8	16.7	19.4	7.3	11.7	12.1
AST(5~40 U/L)	36	34	38	39	32	40	35.3
GGT(5~50 U/L)	88	62	39	18	13	15	16
Tbil(2~19 μmol/L)	120.3	85	64.1	55	43.1	41	26
Dbil(0~6.8 μmol/L)	61.3	46.8	37.3	35.1	25.7	22.5	13.2
Ibil(1.7~13 μmol/L)	59	38.2	26.8	19.9	17.4	18.5	12.8
TBA(0~10μmol/L)	12.7	25.8	30.2	50	20.8	22.8	15.3

TBil, total bilirubin; DBil, direct bilirubin; IBil, indirect bilirubin; GGT, γ-glutamyl transferase; TBA, total bile acids; UDCA, ursodeoxycholic acid; DJS, Dubin-Johnson syndrome.

**Figure 2:**
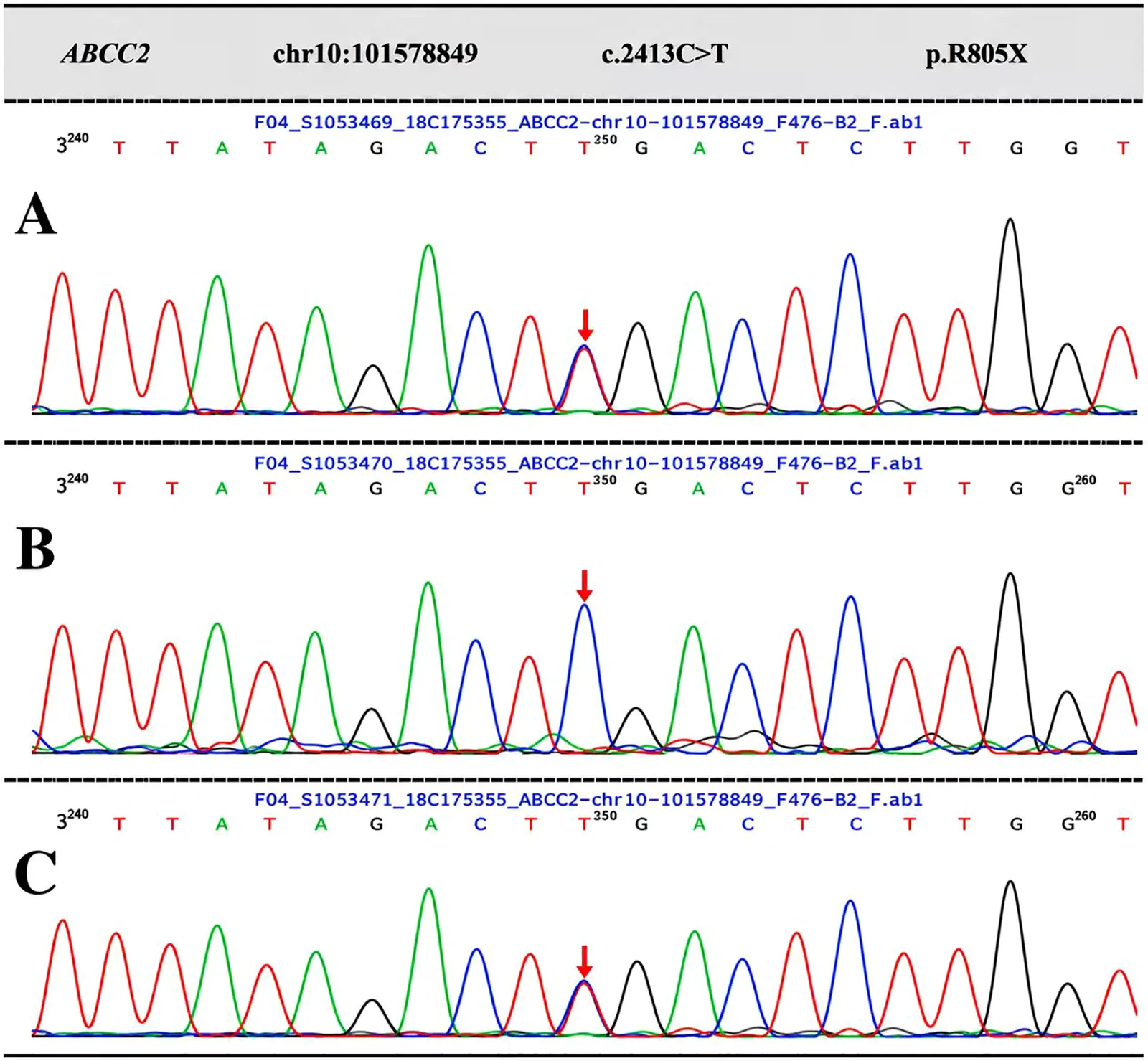
Sanger sequencing validation of the *ABCC2* gene mutations in the proband and her parents. **(A)** Sequencing chromatograms showing the c.2443C>T (p.R815X) mutation in the proband. The arrow indicates the heterozygous C>T transition. **(B)** Sequencing chromatograms of the proband’s mother at the same locus, demonstrating a heterozygous C>T mutation (carrier status). **(C)** Sequencing chromatograms of the proband’s father at the same locus, showing a wild-type sequence without the mutation. The reference sequence is shown above each chromatogram.

**Figure 3:**
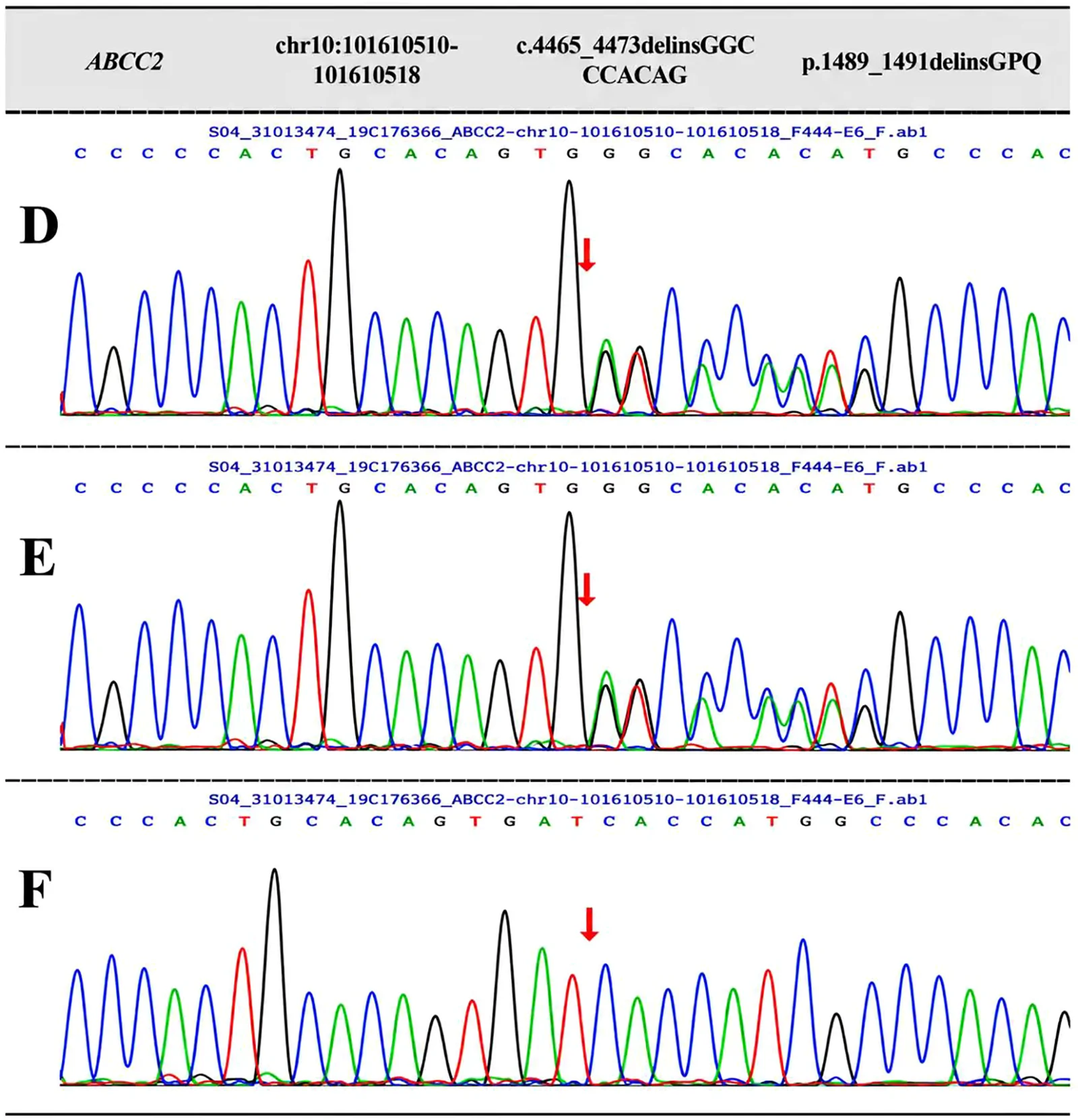
Sanger sequencing validation of the *ABCC2* c.4465_4473delinsGGCCCACAG (p.1489_1491delinsGPQ) mutation in the proband and her parents. **(D)** Sequencing chromatograms of the proband showing the heterozygous deletion-insertion mutation (c.4465_4473delinsGGCCCACAG). The arrow indicates the altered sequence region. **(E)** Sequencing chromatograms of the proband’s father at the same locus, demonstrating a heterozygous mutation (carrier status). **(F)** Sequencing chromatograms of the proband’s mother at the same locus, showing a wild-type sequence without the mutation. The reference sequence is shown above each chromatogram.

**Figure 4:**
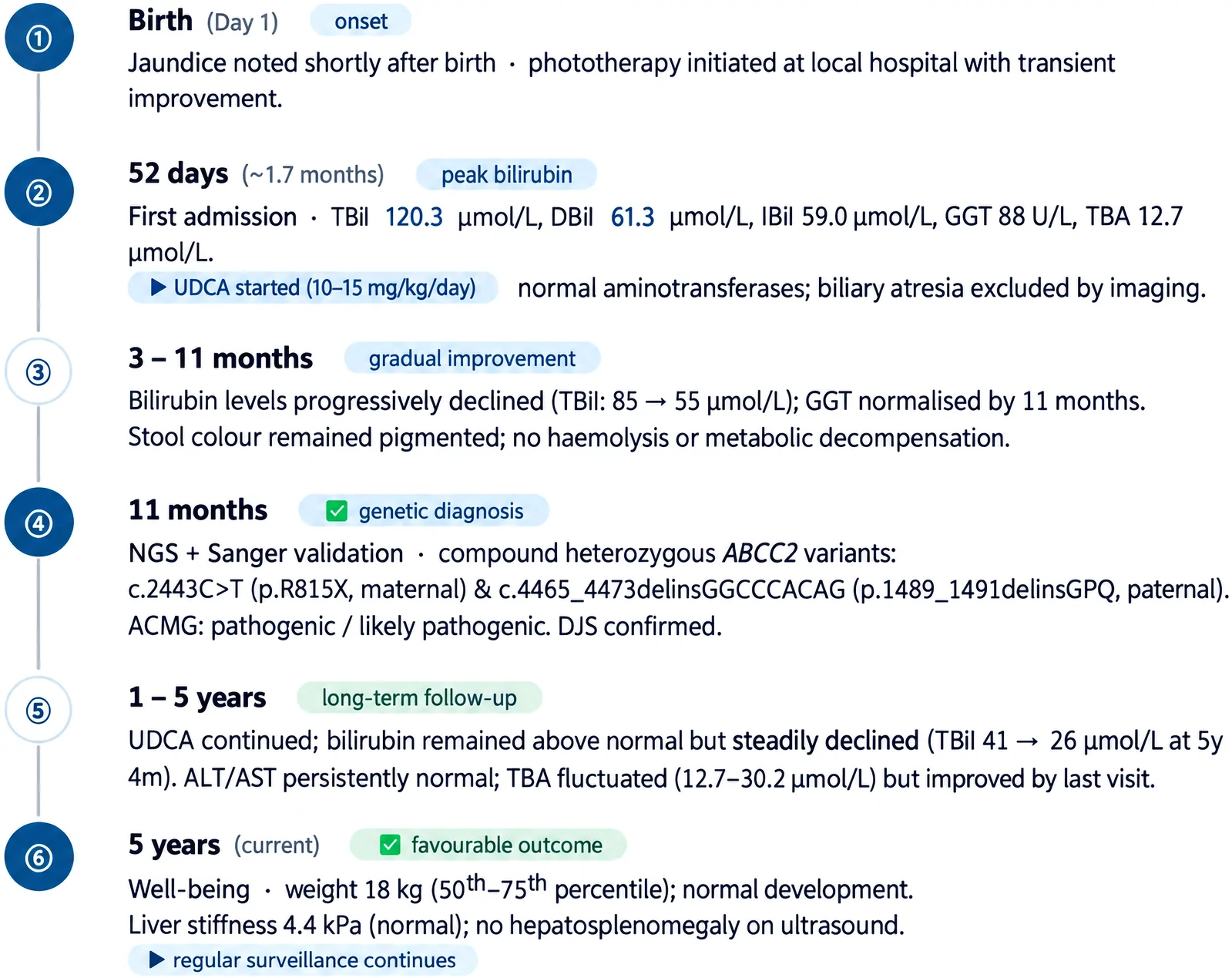
Clinical timeline patient with neonatal-onset Dubin-Johnson syndrome.

## Discussion

The present report describes a case of neonatal-onset DJS caused by compound heterozygous variants in the ABCC2 gene, namely, c.2443C>T (p.R815X) and c.4465_4473delinsGGCCCACAG (p.1489_1491delinsGPQ). A search of the Human Gene Mutation Database and review of the literature revealed that the c.4465_4473delinsGGCCCACAG variant was recently reported by Zhao C et al., whereas the c.2443C>T (p.R815X) variant has not been previously described (5). Thus, the primary contribution of this case is the expansion of the ABCC2 mutational spectrum with the novel c.2443C>T variant, presented in a compound heterozygous combination with a known pathogenic variant, in a patient with infantile-onset disease. The patient presented shortly after birth with predominantly conjugated hyperbilirubinemia, while serum aminotransferase levels remained consistently within reference ranges and no hepatosplenomegaly was detected. Following treatment with UDCA, bilirubin levels gradually declined over time. This case expands the known mutational spectrum of *ABCC2* and further highlights the value of genetic testing for the early diagnosis of neonatal-onset DJS.

Traditionally, DJS is regarded as a disorder that predominantly manifests during adolescence, with neonatal and infantile presentation considered relatively uncommon. However, emerging evidence indicates that clinically significant disease may emerge during the early postnatal period in a subset of affected individuals (2, 3). In the present case, the patient exhibited marked hyperbilirubinemia at 52 days of age, with a total bilirubin concentration of 120.3 μmol/L and a direct bilirubin concentration of 61.3 μmol/L, consistent with neonatal-onset DJS. In contrast to the typical presentation in adult patients, neonatal DJS often manifests as more pronounced cholestasis and may therefore be misdiagnosed as severe cholestatic disorders such as biliary atresia or PFIC, potentially leading to unnecessary surgical exploration (2). In our patient, imaging studies revealed no structural abnormalities of the biliary tract, and GGT levels gradually returned to the reference range during follow-up. Additionally, the stool color remained consistently pigmented throughout the observation period, which further supported the exclusion of biliary atresia (typically characterized by acholic or pale stools). These findings were helpful in distinguishing DJS from biliary atresia (characterized by persistent marked GGT elevation and acholic stools) and PFIC (generally characterized by progressive liver injury accompanied by abnormal aminotransferase levels). Notably, the melanin-like pigment deposition in liver tissue is identified in only approximately 38% of neonatal DJS cases (2). This suggests a relatively high likelihood of missed or delayed diagnoses due to sole reliance on pathological findings. Given these limitations, genetic testing has become the “gold standard” for establishing a definitive diagnosis of DJS (5, 6). Viñuela M et al. (7) described a patient with DJS in whom a striking “bright-blue” liver was observed during surgery. Subsequent genetic analysis identified the *ABCC2* variants c.2077G>A and a novel c.513del mutation, demonstrating the diverse manifestations associated with DJS. Likewise, Gao F et al. (8) performed genetic screening in 56 infants with unexplained jaundice and achieved a diagnostic yield of 51.8%. Among these cases, one patient was diagnosed with coexisting DJS and Rotor syndrome. The distinction between DJS and Rotor syndrome is clinically important, as both conditions present with predominantly conjugated hyperbilirubinemia. In our patient, targeted sequencing revealed no pathogenic variants in SLCO1B1 or SLCO1B3, effectively excluding Rotor syndrome. Furthermore, the serum total bile acid level at initial presentation was only mildly elevated (12.7 μmol/L), which is consistent with the characteristic biochemical profile of DJS, whereas Rotor syndrome typically demonstrates more marked bile acid retention. Urinary coproporphyrin analysis, which was not performed in this case, would have provided additional discriminatory information, as DJS is characterized by normal total urinary coproporphyrin levels with a markedly increased proportion of coproporphyrin isomer I (typically >80% of total), while Rotor syndrome shows elevated total urinary coproporphyrin excretion (approximately 2.5- to 5-fold above normal) with a less prominent increase in the isomer I ratio. Their findings further support the inclusion of DJS in the differential diagnosis of infants presenting with unexplained jaundice. The finding of elevated indirect bilirubin (59.0 μmol/L) at initial presentation warrants brief comment. While DJS is characterized by predominantly conjugated hyperbilirubinemia, a mixed pattern can occur in neonatal patients due to the superimposition of physiological jaundice and increased bilirubin load from hemolysis of fetal hemoglobin. In our patient, a hemolysis screen (reticulocyte count and peripheral blood film) was unremarkable and Coombs testing was negative, effectively excluding a primary hemolytic process. The indirect hyperbilirubinemia was therefore considered to reflect the expected physiological component in a 52-day-old infant, rather than a coexisting pathological condition. This mixed pattern should not detract from the diagnosis when the conjugated fraction remains disproportionately elevated and other diagnostic criteria are met.

In the present case, NGS followed by Sanger sequencing successfully established the parental origin of both variants, and their pathogenicity was evaluated according to the ACMG guidelines using the evidence codes detailed in the Case presentation section. The c.2443C>T variant is a nonsense mutation predicted to introduce a premature termination codon, thereby truncating MRP2 protein synthesis. In contrast, c.4465_4473delinsGGCCCACAG represents an in-frame mutation that may alter the local conformation of the second nucleotide-binding domain (NBD2). The mutational spectrum of *ABCC2* is highly heterogeneous and encompasses missense, nonsense, splice-site, deletion, and insertion variants (5, 6, 9). Demir E et al. (6) applied NGS to 14 children with cholestasis who remained undiagnosed after conventional clinical evaluation and achieved a diagnostic yield of 85.7%. Among these patients, four were diagnosed with DJS, including one carrying a previously unreported homozygous frameshift variant, c.4246_4247del. Similarly, Sun RY et al. (9) reported a DJS proband harboring compound heterozygous variants c.1177C>T (p.R393W) and c.3632T>C (p.L1211P). *In vitro* experiments demonstrated that p.R393W accelerated MRP2 degradation through the ubiquitin–proteasome pathway, whereas p.L1211P reduced MRP2 expression at the plasma membrane. Zhao C et al. (5) identified three novel pathogenic variants in two unrelated DJS families. Notably, one of these variants, c.4465_4473delinsGGCCCACAG, was identical to the paternally inherited variant detected in our patient, providing additional evidence supporting its pathogenic role. The previously reported carrier was an 18-year-old male, whereas our patient is a female with disease onset during infancy. This observation suggests that the same pathogenic variant may give rise to a broadly similar clinical phenotype across different sexes, while the age at disease onset may vary considerably.

At the molecular level, the *ABCC2* gene encodes MRP2, an ATP-dependent transporter localized to the canalicular membrane of hepatocytes, where it mediates the biliary excretion of conjugated bilirubin (10). Mao YX et al. (10) resolved three distinct conformational states of human MRP2 and identified a unique regulatory (R) domain. In the resting state, the R domain occupies the transport cavity and functions as an affinity filter. Upon substrate binding, the R domain is displaced from the cavity, whereas ATP hydrolysis induces interactions between the C-terminal segment of the R domain and transmembrane helices, thereby stabilizing the outward-facing conformation required for substrate export. In the present case, the nonsense variant. In the present case, the nonsense variant c.2443C>T is predicted to disrupt the structural integrity of the R domain or nucleotide-binding domains, although this mechanistic inference is speculative in the absence of functional studies. Similarly, the in-frame variant c.4465_4473delinsGGCCCACAG is located within the NBD2 region and might theoretically impair ATP hydrolysis efficiency, but this remains to be confirmed experimentally. Consistently, Sun R et al. (11) demonstrated that the splice-site variant c.2439 + 5G>A induced skipping of exon 18, leading to the deletion of 56 amino acids within NBD1. This structural alteration markedly reduced MRP2 expression and abolished transporter function. Zhao D et al. (12)noted that DJS caused by homozygous or compound heterozygous *ABCC2* variants generally follows a benign clinical course and rarely progresses to cirrhosis. Nevertheless, careful consideration of drug toxicity is essential in DJS management.

The patient carried a maternally inherited nonsense variant and a paternally inherited in-frame variant, resulting in a compound heterozygous genotype. Biallelic loss-of-function variants in *ABCC2* are generally expected to cause substantial impairment of MRP2 function. Despite this genetic background, the clinical course in our patient was relatively mild. Although conjugated hyperbilirubinemia was prominent during the neonatal period, serum aminotransferase levels remained consistently normal throughout follow-up. Moreover, bilirubin concentrations gradually declined with age, and no evidence of hepatic fibrosis or biliary cirrhosis has been observed to date. These findings are in agreement with the natural history described in previous studies of neonatal-onset DJS (2, 3). Kim JH et al. (13) reported a 50-year-old woman with DJS in whom a black-pigmented liver was identified during cholecystectomy. Genetic analysis revealed a missense variant, R768W, in exon 18 of *ABCC2*, and immunohistochemical staining demonstrated near-complete loss of MRP2 expression. Nevertheless, the patient exhibited only mild conjugated hyperbilirubinemia without evidence of progressive liver injury. In contrast, Long J et al. (2) described a neonatal case developing jaundice on the sixth day of life, with direct bilirubin levels rising progressively to 260 μmol/L. The patient ultimately underwent laparoscopic cholecystostomy followed by medical treatment, after which bilirubin levels normalized. Compared with that case, the disease severity in our patient was considerably milder. A multicenter retrospective study conducted by Hasosah M et al. (14) included 11 cases of neonatal DJS. All patients presented primarily with conjugated hyperbilirubinemia, while GGT levels were within or only mildly above reference ranges. Importantly, none developed progressive liver injury during follow-up. These observations provide further evidence that in neonates, DJS is generally associated with favorable outcomes. In most cases, DJS does not require disease-specific treatment (3, 5). However, in neonates presenting with severe cholestasis, therapeutic intervention may help reduce intrahepatic pigment accumulation and alleviate biochemical abnormalities (2). In the present case, ursodeoxycholic acid therapy was continued after the diagnosis was established, and serum bilirubin levels decreased substantially within one month. However, it is important to acknowledge that this biochemical improvement coincided with the natural trajectory of the disease, as spontaneous decline in bilirubin levels with age has been well documented in DJS. Given the observational nature of this single case report and the absence of a treatment-free period for comparison, the specific contribution of UDCA to the observed reduction cannot be definitively determined. Zhang Y et al. (3) described a 19-day-old female infant carrying compound heterozygous variants c.4237_4238insCT and c.517G>A. Following treatment with UDCA and a short course of phenobarbital, both bilirubin and liver enzyme levels gradually normalized during a two-year follow-up period. Likewise, Siddiqui AH et al. (15) reported a 17-year-old male patient whose conjugated bilirubin concentration decreased from 40 μmol/L to 29 μmol/L after treatment with UDCA combined with rifampicin. Lee JH et al. (16) followed four patients diagnosed with DJS during the neonatal period for 5–20 years and observed a second peak of jaundice during adolescence in one individual. This finding suggests that patients with neonatal-onset DJS should undergo long-term surveillance.

The clinical significance of this case lies in several aspects. First, it expands the mutational spectrum of *ABCC2* by identifying two previously unreported variants. Notably, the c.4465_4473delinsGGCCCACAG variant is identical to that reported by Zhao C et al. (5), suggesting that this variant may be more prevalent than currently recognized. Second, the present case highlights the importance of early genetic testing in neonates presenting with unexplained conjugated hyperbilirubinemia and persistently normal aminotransferase levels, which may help avoid unnecessary invasive investigations. Third, the favorable outcomes observed in this patient further support the effectiveness of conservative treatment in neonatal-onset DJS. Several limitations of this study should be acknowledged. First, formal parentage testing was not performed. Second, urinary coproporphyrin analysis was not performed, which has been suggested as a useful non-invasive diagnostic adjunct for DJS in some recent studies. Third, liver biopsy was declined by the patient’s family, precluding pathological evaluation and immunohistochemical assessment of MRP2 expression. Fourth, clinical information from the period preceding 52 days of age was incomplete. Finally, without *in vitro* functional studies, potential pathogenic mechanisms were inferred primarily from the ACMG guidelines and comparisons with existing literature.

## Conclusion

In conclusion, this report describes a case of neonatal-onset DJS caused by compound heterozygous ABCC2 variants, including a novel c.2443C>T (p.R815X) variant and a recently reported c.4465_4473delinsGGCCCACAG (p.1489_1491delinsGPQ) variant. The diagnosis was established through genetic testing, and the patient showed a favorable clinical course. This case expands the mutational spectrum of ABCC2 with a novel variant and underscores the central role of genetic testing in the diagnosis of neonatal-onset DJS.

## Data Availability

The raw data supporting the conclusions of this article will be made available by the authors, without undue reservation.
